# An extravascular fluid transport system based on structural framework of fibrous connective tissues in human body

**DOI:** 10.1111/cpr.12667

**Published:** 2019-08-01

**Authors:** Hongyi Li, Chongqing Yang, Yajun Yin, Fang Wang, Min Chen, Liang Xu, Naili Wang, Di Zhang, Xiaoxia Wang, Yiya Kong, Qing Li, Si Su, Yupeng Cao, Wentao Liu, Zhuo Ao, Luru Dai, Chao Ma, Lijun Shang, Dong Han, Fusui Ji, Hua Li

**Affiliations:** ^1^ Beijing Hospital National Center of Gerontology Beijing China; ^2^ Department of Engineering Mechanics Tsinghua University Beijing China; ^3^ Department of Human Anatomy, Histology and Embryology, Neuroscience Center, Institute of Basic Medical Sciences Chinese Academy of Medical Sciences Beijing China; ^4^ School of Basic Medicine Peking Union Medical College Beijing China; ^5^ National Center for Nanoscience and Technology Beijing China; ^6^ School of Chemistry and Biosciences University of Bradford Bradford UK; ^7^ Institute of Computing Technology Chinese Academy of Sciences Beijing China

**Keywords:** extracellular matrix, fibrous connective tissues, gross anatomy, interfacial transport, interstitial fluid

## Abstract

**Objective:**

Interstitial fluid in extracellular matrices may not be totally fixed but partially flow through long‐distance oriented fibrous connective tissues via physical mechanisms. We hypothesized there is a long‐distance interstitial fluid transport network beyond vascular circulations.

**Materials and methods:**

We first used 20 volunteers to determine hypodermic entrant points to visualize long‐distance extravascular pathway by MRI. We then investigated the extravascular pathways initiating from the point of thumb in cadavers by chest compressor. The distributions and structures of long‐distance pathways from extremity ending to associated visceral structures were identified.

**Results:**

Using fluorescent tracer, the pathways from right thumb to right atrium wall near chest were visualized in seven of 10 subjects. The cutaneous pathways were found in dermic, hypodermic and fascial tissues of hand and forearm. The perivascular pathways were along the veins of arm, axillary sheath, superior vena cava and into the superficial tissues on right atrium. Histological and micro‐CT data showed these pathways were neither blood nor lymphatic vessels but long‐distance oriented fibrous matrices, which contained the longitudinally assembled micro‐scale fibres consistently from thumb to superficial tissues on right atrium.

**Conclusions:**

These data revealed the structural framework of the fibrous extracellular matrices in oriented fibrous connective tissues was of the long‐distance assembled fibres throughout human body. Along fibres, interstitial fluid can systemically transport by certain driving‐transfer mechanisms beyond vascular circulations.

## 1 INTRODUCTION

The earliest record of the long‐distance extravascular fluid flow was the perivascular spaces in brain around 1850s. However, fluid in the interstitial matrices is thought to be mainly entrapped locally by proteoglycan filaments^1^ and local fluid in the distal end of human extremities is retaken only by venous and lymphatic vessels. Whether fluid in the interstitial matrix can flow systemically throughout the body beyond vascular circulations has been debated for decades.^2^, ^3^ Recently, several studies showed that partial interstitial fluid can flow along tunica adventitia or through a macroscopic fluid‐filled interstitial space, forming an extravascular transport pathway for interstitial fluid.^2^, ^3^, ^4^

Starting from 2006, we focused on a long‐distance transport phenomena of interstitial fluid in animal and humans and found two types of long‐distance extravascular fluid transport pathways, a cutaneous and a perivascular pathway.^4^, ^5^ For example, in rabbit, extravascular fluid transport from the lower limb into pericardial cavity was found to be via the venous adventitia and its surrounding fibrous connective tissues.^4^

In one amputee of lower leg patient, who received the hypodermic injection of the fluorescent tracer into ankle dermis before taking an amputation of lower leg due to severe gangrene of foot, extravascular fluid transport from ankle dermis to the broken end of lower leg was anatomically identified to be the cutaneous pathway (located in dermis and the interlobular septum among hypodermic adipose tissues) and the perivascular pathway (located in general connective tissues surrounding the veins and the arteries), and both pathways were composed of the oriented fibrous connective tissues.^5^ By two‐photon confocal laser microscopy (TPCLSM), the longitudinal fibres along the transport long‐axis of these pathways were stained by the fluorescein from ankle.^5^ However, fibrous interstitial matrices are usually considered to be a three‐dimensional architecture with the isotropic fibres. The reason that why only the fibres towards the transport long‐axis were stained by the fluorescein was unclear.

In healthy volunteers, two peripheral long‐distance extravascular fluid transport pathways originating from acupuncture points in the extremity endings were also found by MRI.^5^, ^6^, ^7^ Our experiments showed there were two types of “the entrant points or hypodermic entrances” for the tracer injection to visualize the long‐distance extravascular pathways of the extremities.^5^, ^6^ One was a hypodermic point in the vicinity of venous vessels of the forearm or lower legs, which was used to visualize the perivascular pathways. The other was a hypodermic point at the distal end of the fingers or toes or around the wrist or ankle, which was usually an acupuncture point and applied to visualize both the cutaneous and perivascular pathways. However, the comprehensive distributions of the long‐distance extravascular pathways have not been fully understood due to the unfitness of current contrast‐enhanced MRI technique in tracing the paramagnetic agent in the visceral organs of volunteers. The connections of the peripheral extravascular pathways with the associated visceral structures were still unknown in human.

Moreover, both the kinetic/dynamic mechanism and regulating factors for these long‐distance extravascular fluid transport through fibrous matrices remain unknown. For fibrous matrix, neither the collagenous and elastic fibres nor the gel‐like substances can flow freely.^1^ The demonstrated staining processes of the fluorescent fluid along the fibres suggested that an interfacial transport zone between the solid fibre and the surrounding gel‐like substances could be responsible for the fluid transport.^5^ As to regulating factors, our pilot study in the amputated legs revealed that the mechanical force is one of dynamic origins to “pull or push” fluid from the extravascular pathways.^5^ Our study in rabbits showed the peripheral extravascular fluid from ankle was transported along the perivenous fibrous tissues surrounding the inferior vena cava, three grooves of the heart and into the pericardial cavity to form pericardial fluid.^4^ Thus, we speculate the mechanical heart beatings might be one of the regulating factors to drive fluid through the oriented fibrous matrices of connective tissues in physiological conditions.

In current study, we first used twenty healthy volunteers to further demonstrate two types of the hypodermic entrant points by MRI where are acupuncture points in the extremity endings. We then chose one of the entrances identified in healthy volunteers as the hypodermic injection point in human cadavers. To visualize the long‐distance extravascular pathways, we used a mechanical chest compressor device to simulate the heart beatings to drive interstitial fluid flow.^8^ The anatomic distributions and histological structures of extravascular pathways from the extremity ending to the associated visceral structures were consequently identified. By the current experiment of dynamic gross anatomy, we may verify the structural framework of the oriented long‐distance extravascular fluid transport pathways throughout human body and the hypothesis there is a long‐distance interstitial fluid transport network composed of oriented fibrous matrices of connective tissues beyond vascular circulations.

## 2 METHODS

### 2.1 MRI image of the pathways from acupuncture points of hand and foot in volunteers

Twenty healthy volunteers aged from 23 to 37 years (15 men and five women) were recruited. Exclusion criteria included a history of primary or secondary extremity lymphedema, obesity (lipoedema), extensive scarring or dermatological abnormalities in the areas tested and contraindications for MRI. The study was approved by the ethics committee of Beijing Hospital (No. 2016BJYYEC‐066‐02). All the participants have signed the informed consent prior to the initiation of the study.

**Figure 1:**
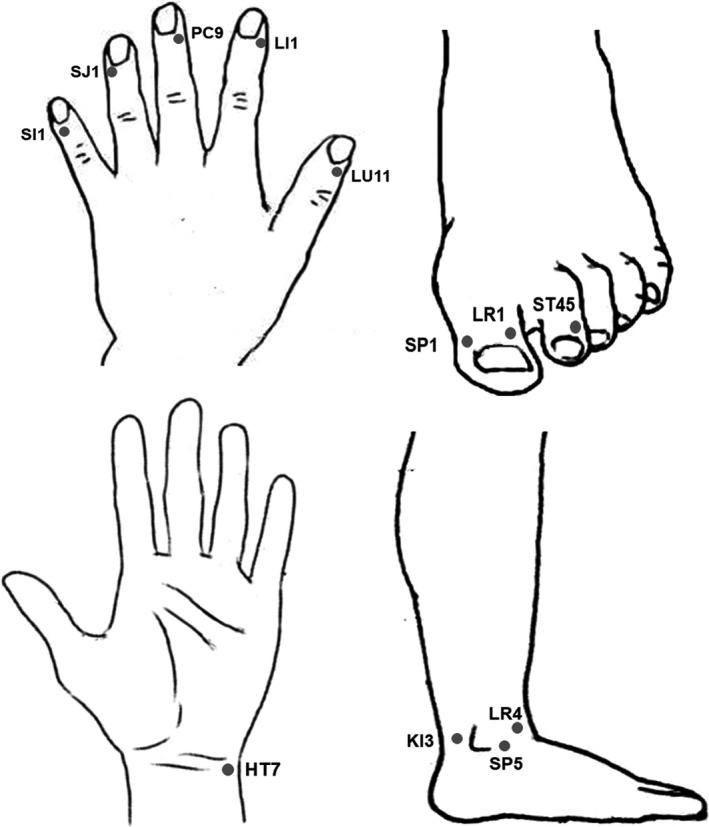
Illustration of the hypodermic injection points in hand and foot. The positions of each point were listed in Table 1

Five Jing‐well points on fingers, an acupuncture point on wrist, three Jing‐well points on toes and three acupuncture points on ankle were selected as the hypodermic injection points for MRI. Details of injection points, the locations and the corresponding acupuncture points were listed in Table 1 and Figure 1.

**Table 1:** Injection points, their physiological positions and the corresponding acupuncture points

Injection point	Physiological location	Acupuncture point
Distal end of thumb	Posterior to the thumb nail on the radial side	Shao Shang (LU11) of the Lung Meridian
Distal end of index finger	Posterior to the corner of the nail on the radial side of the index finger	Shang Yang (LI1) of the Large Intestine Meridian
Distal end of middle finger	In the centre of the tip of the middle finger	Zhongchong (PC9) of the Pericardium Meridian
Distal end of ring finger	Posterior to the Ring fingernail on the ulnar side	Guanchong (SJ1) of the Sanjiao Meridian
Distal end of little finger	Posterior to the little fingernail on the ulnar side	Shaoze (SI1) of the Small intestine Meridian
The point at wrist	At the wrist crease, on the radial side of the flexor carpi ulnar tendon	Shenmen (HT7) of the Heart Meridian
Distal end of medial side of great toe	Posterior to the corner of the nail, on the medial side of the great toe	Yinbai (SP1) of the Spleen Meridian
Distal end of lateral side of great toe	On the lateral side of the great toenail	Dadun (LR1) of the Liver Meridian
Distal end of 2nd toe	Posterior to the corner of the nail on the lateral side of the 2nd toe of foot	Li Dui (ST45) of the Stomach Meridian
The 1st point at ankle	In depression midway between the tip of the medial malleolus and the attachment of the achilles tendon, level with the tip of the medial malleolus	Taixi (KI3) of the Kidney Meridian
The 2nd point at ankle	Anterior to the medial malleolus, in a depression on the medial side of the tendon of tibialis anterior	Zhongfen (LR4) of the Liver Meridian
The 3rd point at ankle	In a depression distal and inferior to the medial malleolus, midway between the tuberosity of the navicular bone and the tip of the medial malleolus	Shangqiu (SP5) of the Spleen Meridian

Each injection point was tested on two volunteers. The choice of right or left hand or foot depended on the will and preference of the volunteers. Two volunteers received the routine MR angiography on forearm. After the angiography, an injection point, which was near a superficial vein and neither acupuncture point nor a point on Meridians, was selected as the control.

The depth of the injection was approximately 1‐2 mm into subcutaneous loose connective tissues of the point. The paramagnetic contrast agent, gadolinium diethylenetriamine penta‐acetic acid (Gd‐DTPA, Magnevist; Bayer Schering Pharma AG), was hypodermically administered at minimal volumes of 0.2‐0.5 mL (the final diluted concentration was 156 mg/mL). The transport pathways originating from the acupuncture points were scanned by an Achieva 3.0T TX scanner (Philips Electronics) or 1.5T MR Scanner (GE Medical Systems). The images were obtained using a 3‐dimensional T1‐weighted spoiled gradient‐echo sequence (SPGR) or fast field echo (FFE) sequence using an 8‐channel phased‐array head coil. Scanning parameters were adjusted to obtain increased spatial resolution. The raw data were analysed using an extended MR WorkSpace station with maximum intensity projection reconstructions (MIPs).

### 2.2 General medical information of the donated human cadavers

The human cadaver experiment was supported by the Human Tissue Bank, Chinese Academy of Medical Sciences & Peking Union Medical College, which was approved by the Institutional Review Board of the Institute of Basic Medical Sciences, Chinese Academy of Medical Sciences (Approval Number: 009‐2014). The research protocol was approved by the ethics committee of Beijing Hospital (No. 2013BJYYEC‐037‐02). Nineteen frozen human cadavers but not preserved in formalin solution were selected from October 2016 to March 2019. Each subject was left unfrozen at room temperature before the experiments. The general information was as below in Table 2.

**Table 2:** General medical information of the donated human cadavers

Subject	Gender & age	Main death conditions	Preservation time before unfrozen
1	F/92	Pneumonia	72 h
2	M/74	Lung cancer	48 h
3	M/92	Oesophageal carcinoma	2 wk
4	M/86	Lung cancer	3 mo
5	F/86	Colorectal cancer	2 wk
6	M/95	Pneumonia	1 wk
7	M/68	Oesophageal carcinoma with severe cachexia	3 wk
8	F/77	Gastric carcinoma with severe cachexia	8 wk
9	M/76	Lung cancer	7 wk
10	F/78	Ovarian cancer with severe cachexia	7 wk
11	M/49	Cerebral haemorrhage	5 wk
12	F/35	Ovarian cancer	72 h
13	M/7	Myocarditis	1.5 y
14	M/73	Lung cancer	10 wk
15	M/79	Lung cancer	2 wk
16	M/83	Myocardial infarction	1 wk
17	M/89	Pneumonia	3 wk
18	M/68	Oesophageal carcinoma	2 wk
19	F/87	Lung cancer	1 wk

### 2.3 Hypodermic injection on the right thumb and the visualization of the long‐distance extravascular fluid transport pathway in cadavers

To visualize the long‐distance fluid transport pathway, the distal end of right thumb was selected as the injection point in this study. 0.3 mL of fluorescein sodium solution (Fluorescein, final diluted concentration was 10 g/L by natural saline; WuZhou Zhongheng Group Co., Ltd) was injected into the hypodermis with a depth of 1‐2 mm by 1 mL syringe (B. Braun Medical). The injection method of paramagnetic contrast, Gadopentetate Acid Dimeglumine (Gd‐DTPA, Magnevist; Schering China Limited), and the Chinese ink was as the same as those of fluorescein.

Ten cadaver subjects (No. 1‐10) were injected by fluorescein. The movements of the fluorescein in the 10 subjects were observed for 2.5 hours before the next experiments. Secondly, continuous chest compressions were performed right on the heart for the subsequent 2.5 hours with the frequency of 110 bpm by an automatic mechanical chest compressor device (Weil MCC100).^8^ The dissection processes were recorded under a blue/violet light with digital camera. Samples from the fluorescently stained and the control were taken for the following studies.

### 2.4 Extravascular fluid transport pathways visualized by MRI

The other three cadaver subjects (No. 11, 12, 13) with Gd‐DTPA (0.5 mL, final diluted concentration of 156 mg/mL) were injected at the same injection point of right thumb and scanned by MRI after the 2.5 hours of repeated chest compressions. The MRI experiments were carried out by 1.5T MR Scanner (GE Medical Systems) using the body coil. The images were obtained using a 3‐dimensional T1‐weighted spoiled gradient‐echo sequence (SPGR). The parameter was adjusted to achieve high image resolution.

### 2.5 Visualization of the lymphatic vessels on hand and forearm

The other two cadaver subjects (No. 14, 15) were given pressure injection with Chinese ink into the same injection point of right thumb as first two groups to visualize lymphatic vessels on hand and forearm. The dissection processes were recorded by digital camera.

### 2.6 Extravascular fluid transport pathways visualized by quantum dot microspheres

The two cadaver subjects (No, 16, 17) were given the injection of 0.5 mL‐10 nm quantum dot microsphere (CdSe/ZnS core/shell quantum dots with carboxylic acid stabilizing ligand; PL: 525 nm ± 5 nm; diameter: 11 ± 1 nm/TEM; Beijing Najing Biological Technology Co., LTD.) into the same point of right thumb. The other two cadaver subjects (No. 18, 19) were given the injection of 0.5 mL‐110 nm quantum dot microsphere (CdSe/ZnS core/shell quantum dot microsphere with carboxylic acid; PL: 565 ± 10 nm; diameter: 110 ± 20 nm/TEM; Beijing Najing Biological Technology Co., LTD.) into the same point of right thumb. After the hypodermic injection, continuous chest compressions were performed right on the heart for the subsequent 2.5 hours with the frequency of 110 bpm by an automatic mechanical chest compressor device. The dissection processes were recorded under a blue/violet light with digital camera.

### 2.7 Histological study and two‐photon laser scanning microscopy examination on the fluorescent pathways

The fluorescently stained tissues on hand, forearm, upper arm, axilla and thorax were sampled, respectively. Sampled tissues were examined histologically by frozen fluorescence, Elastic van Gieson or H&E staining according to standard procedures. Chinese ink was used to permanently locate the exact position of fluorescence in the tissue samples. After the sampling, frozen cross‐sections of 5‐10 μm in thickness were cut from the tissues containing the fluorescent pathways and observed under a fluorescence microscopy. The same slides of the frozen sections were then examined using haematoxylin & eosin (HE) staining and the combined staining methods of Elastic van Gieson (VG), respectively.

The tissues used were the same samples as those of the frozen sections. Two‐photon laser scanning microscopy (TPLSM, Olympus FV1000 and Spectra‐Physics Mai Tai DeepSee) was employed to exam all samples obtained from the skins, and the blood vessels including their surrounding connective tissues. TPLSM was performed at 960 nm for excitation and 495‐630 nm for emission. For each sample, 25 slices were captured using a 0.1‐μm interval at a 512 × 512 resolution.

### 2.8 Immunohistochemical staining on the fluorescent pathways

Immunostaining was performed according to standard procedures. The sections of the fluorescent pathways were immunohistochemically stained with antibodies dilution (1:200) against D2‐40 (Dakocytomation) and antibodies against CD31 (JC/70A; Abcam). The epitope retrieval was performed manually by using pressure cooker. Envision + HRP (Dakocytomation) was subsequently used as secondary detection.

### 2.9 X‐ray 3D micro‐CT on the fluorescent pathways

Firstly, the samples were prepared for decellularization. Tissue samples were treated with 1 mol/L NaCl solution and shaken on a shaker for 24 hours at room temperature. The samples were soaked with n‐Hexane and shaken at room temperature for 24 hours. Secondly, the samples were washed with PBS, soaked in 1% SDS solution and shaken at room temperature for 24 hours. Thirdly, the sample were washed with PBS, soaked in 0.5% trypsin solution and shaken at room temperature for 2.5 hours.

The samples were frozen‐dried for 24 hours and immersed into 10 wt% silver nitrate solution for 30 minutes. 3 wt% sodium hydroxide solution was dropped in slowly with the same volume used for silver nitrate. Half an hour later, the samples were gently washed by deionized water and frozen‐dried again. The prepared samples were preserved in room temperature for the micro‐CT scanning.

The samples were imaged using a high‐resolution 3D X‐ray microcomputed tomography (nanoVoxel‐3000 series; Sanying Precision Instruments Co., Ltd.) according to standard procedures. The parameters were adjusted to obtain higher resolutions. The images were captured on a detector 1920 × 1536 pixels, with an exposure time of 0.6 seconds and a voxel size of 4.6 μm approximately.

## 3 RESULTS

### 3.1 The cutaneous pathways and the perivascular pathways from the hypodermic entrances identified in 20 volunteers by MRI

**Figure 2:**
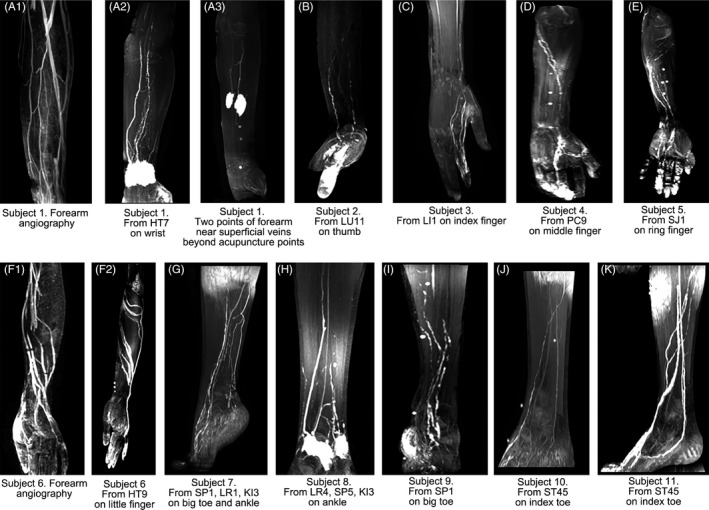
Illustration of the cutaneous pathways and the perivascular pathways originating from the hypodermic injection of hand and foot by MRI. A1, superficial veins in forearm of subject 1. A2, the perivascular pathways and the cutaneous pathways originating from HT7 on wrist of subject 1. By contrast, A3 (Subject 1) showed only the perivascular pathways originated from two hypodermic injection points that were near superficial veins and neither acupuncture point nor a point on Meridians. B, C, D, E and F2, both the cutaneous and perivascular pathways could be visualized by the hypodermic injection into each fingertip of all five fingers in five subjects. F1 showed the superficial veins in the forearm of subject 6 by MRI angiography. By contrast, F2 (subject 6) showed the enhanced pathways partially coincided with the superficial veins of F1 and were partially different from the veins as well. G, H, I, J and K, the same phenomena could be found by the hypodermic injection into the acupuncture point located in toe or ankle of subjects 7‐11

When the injection points were acupuncture points (Table 1, Figure 1), both the cutaneous and the perivascular pathways could be visualized (Figure 2, Videos [Link], [Link], [Link]). If the injection point is near a superficial vein under the skin of the forearm or ankle, only the perivascular pathways were displayed (Figure 2A3).

The cutaneous pathways were located in the hypodermic tissues with an “intraluminal filling defect” appearance (Figure 2A2,B,C,D,E,F2,G,H,I,J,K). The luminal diameter sizes of the cutaneous pathways were approximately 2.0‐8.0 mm when they were measured at different levels of the cross‐sectional planes. The perivascular pathways were the “superficial veins” of forearm or lower leg and exhibited “smooth lumen” (Figure 2A3). These data coincide with our previous findings in volunteers by MRI.^6^, ^7^ In our experiences, it was the venous adventitia that were enhanced by hypodermic Gd‐DTPA other than the venous lumen.

Both the cutaneous pathways and the perivascular pathways were visualized in the distal end of all fingers. Thus, the point of the distal end of the first thumb, which was the Jing‐well point of the Lung Meridian, Shao Shang (LU11) and it is posterior to the thumb nail on the radial side, was selected as the hypodermic injection point in the following experiments.

### 3.2 The fluorescent pathways connecting the thumb with the heart surfaces discovered by gross anatomic methods

Under a blue/violet light and recorded by digital camera, it was clearly shown that only local diffusion of fluorescein occurred around the injection point during the first 2.5 hours in all ten subjects  (No. 1‐10). After the repeated chest compressions for the following 2.5 hours, long‐distance fluorescent pathways were found in seven of ten subjects, which originated from the injection point of thumbs to the superficial tissues of right atrium wall and the pectinate muscles of the right atrial appendage, but not found in the other three subjects with severe cachexia. The reasons could be lack of fluid in interstitium.

**Figure 3:**
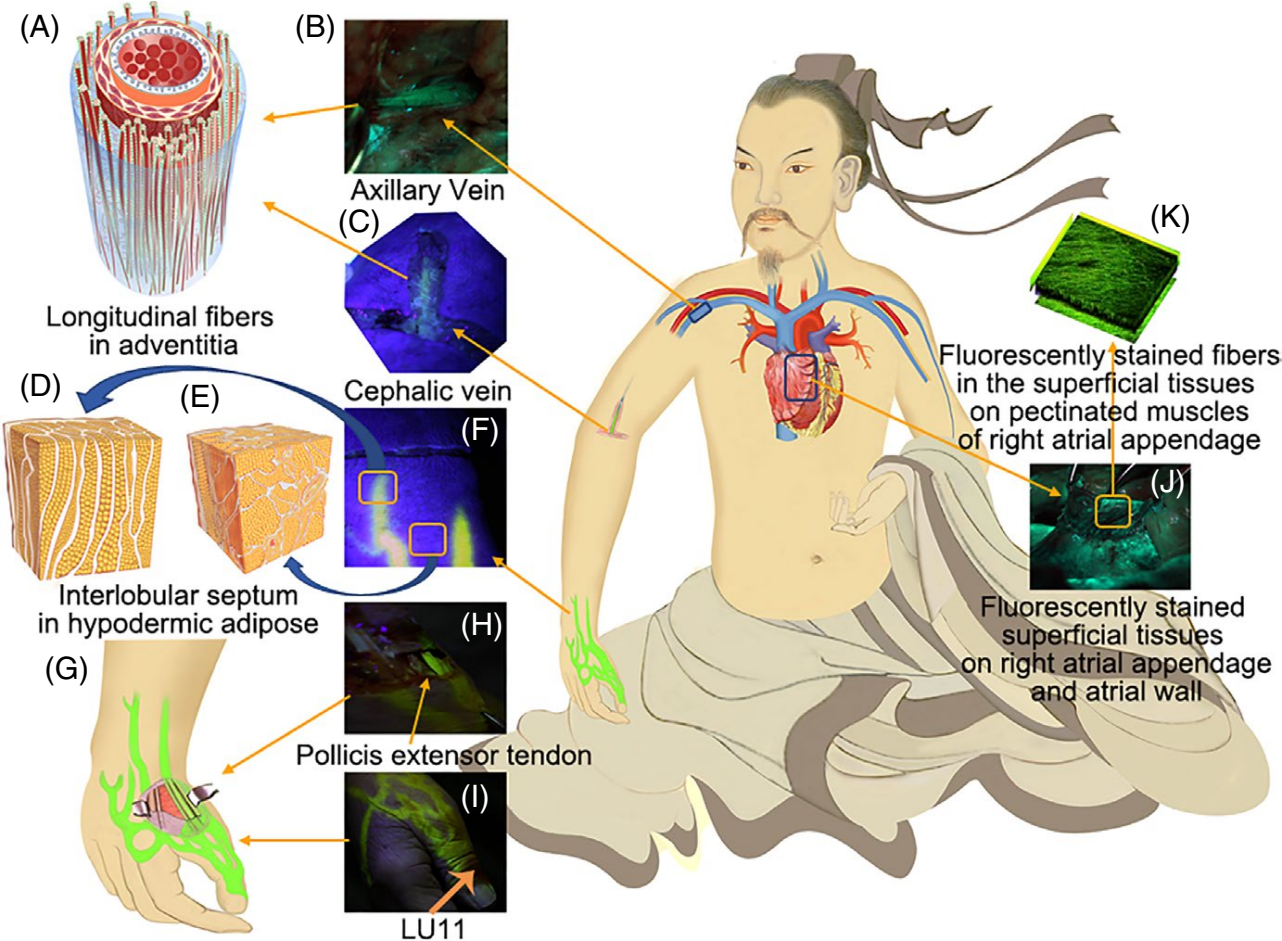
Illustration of the transport courses of the fluorescent pathways from the hypodermic tissues of LU11 into the superficial tissues on right atrium. The photographs were from the 1 cadaver (F/92). By the chest compressions, the injected fluorescein was transported through the cutaneous pathways and the perivascular pathways from the index finger (I), along the wrist (H) and forearm (F), the cephalic venous adventitia and its surrounding tissues (C), the axillary vein (B) and into the superficial tissues on the right atrial wall and pectinated muscles of right atrial appendage (J). The cutaneous pathways of wrist contained diverse structures including the dermis, hypodermis and superficial fascia on muscles or tendons associated with thumb (H). All fluorescent pathways on skin of hand were illustrated at G. The incision of G showed the stained tendons of extensor pollicis brevis, longus and abductor pollicis longus from thumb. By contrast, the tendons from index finger were not stained in G. The interlobular septum of hypodermic adipose was longitudinally towards the long‐axis of the transport pathways (D) and irregular septum (E) beyond the pathways. A, the adventitial fibres were distributed longitudinally along the vessel long‐axis and enriched in one side of the vascular vessel. K, fluorescently stained criss‐crossed fibres in the superficial tissues on pectinated muscles of right appendage by TPLSM

**Figure 4:**
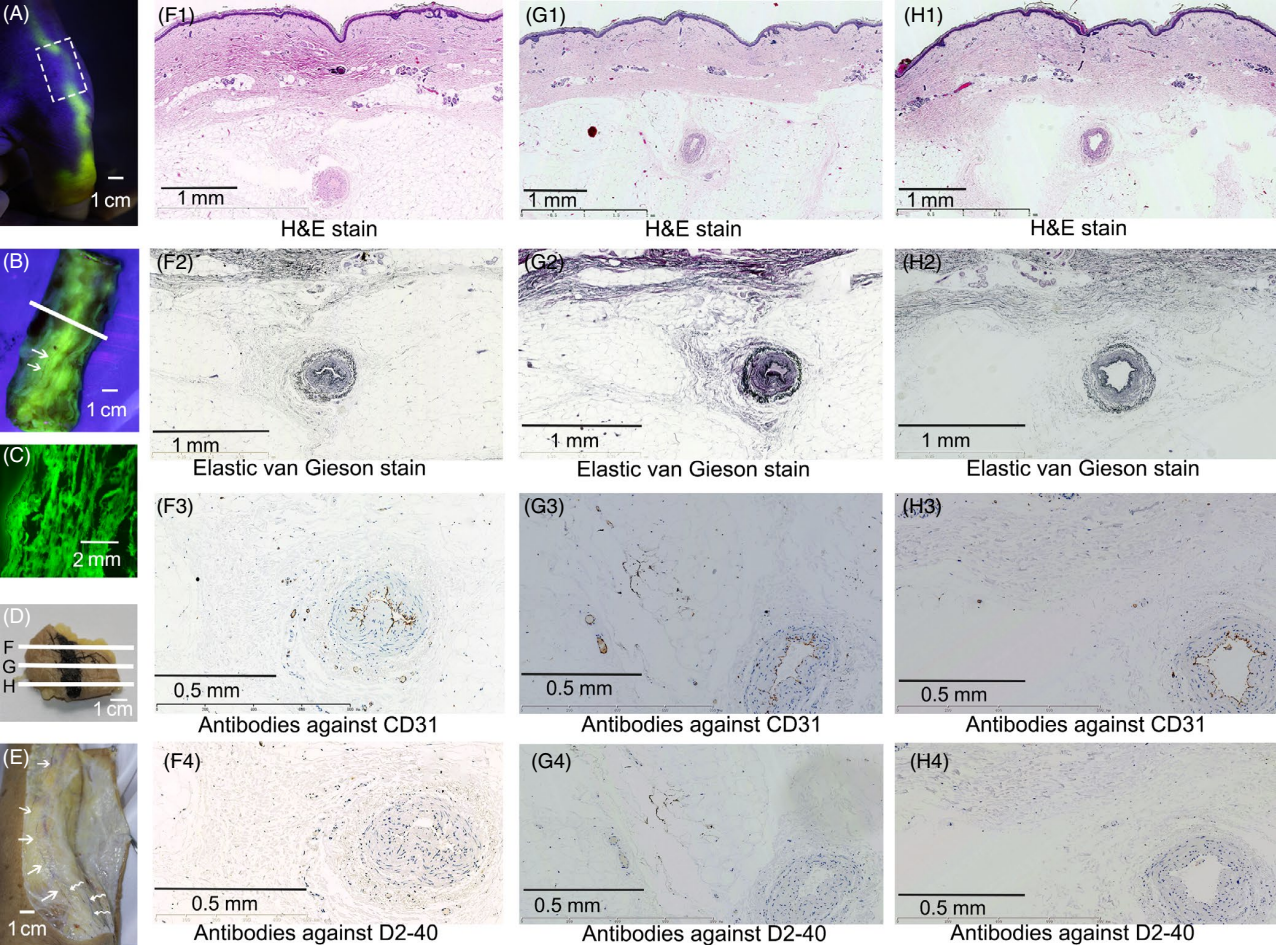
The fluorescent pathways were neither blood nor lymphatic vessels by histological analysis. B showed the internal side of the white box of (A). C, cross‐sectional image of the white line of (B) and showed the fluorescently stained dermic and hypodermic tissues by fluorescence microscopy. The fluorescent pathway on the skin of (D) was painted by Chinese ink. F, G and H, three level of cross‐sections of (D) and analysed histologically by H&E stain, Elastic van Gieson stain and immunochemical stain against CD31 and D2‐40 separately. Except the one small artery (pointed by two white arrows in B) under the skin marked by Chinese ink, there were no continuous blood or lymphatic capillaries found in either F1‐G1‐H1, F2‐G2‐H2, F3‐G3‐H3 or F4‐G4‐H4. E, lymphatic vessels (visualized by pressure injection of Chinese ink) pointed by the straight tail arrows that were different from the superficial veins pointed by curved tail arrows and the fluorescent pathways of (A) and (B)

**Figure 5:**
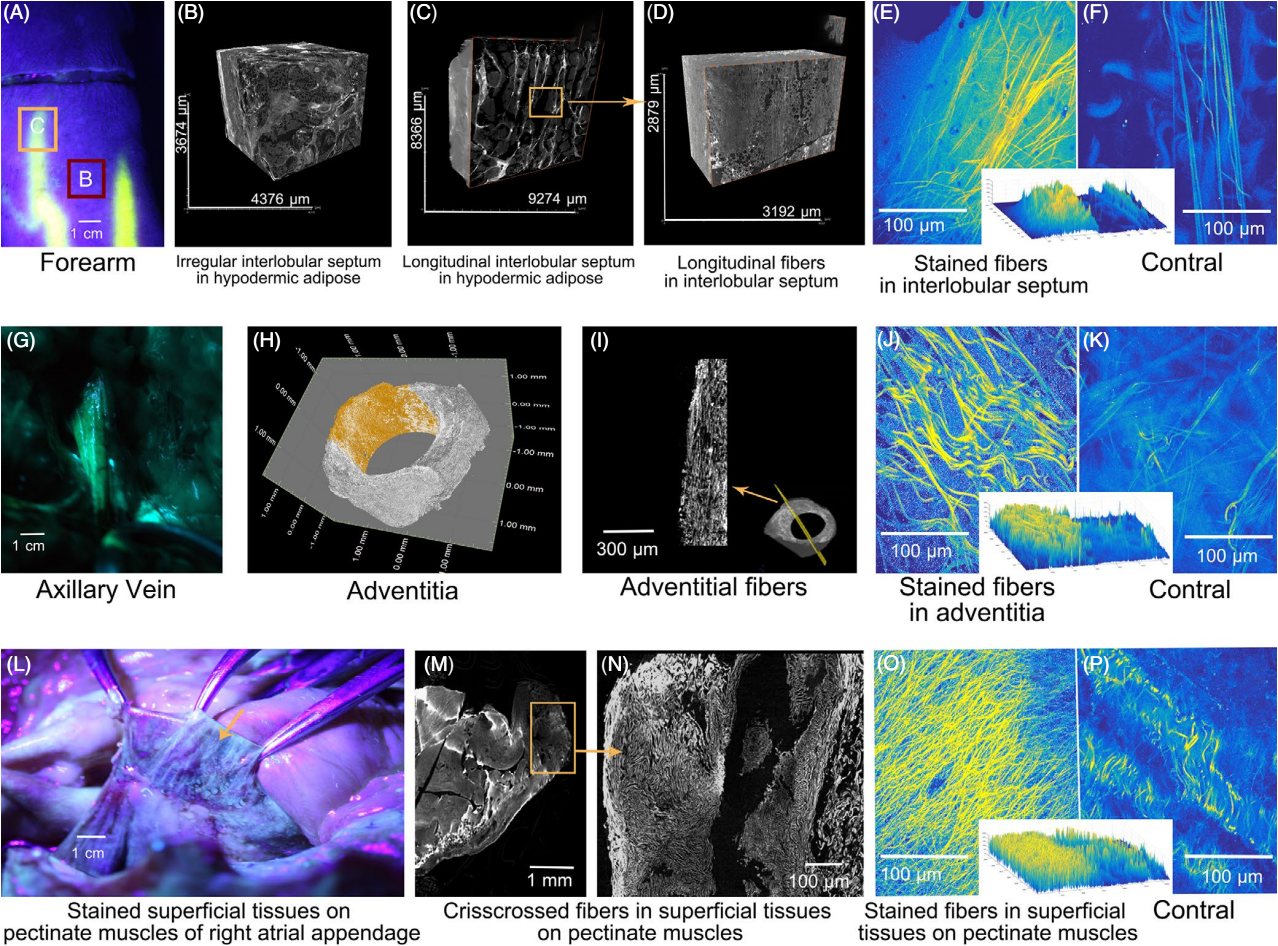
The intrinsic fibres of fibrous connective tissues in different anatomic sites disclosed by micro‐CT and TPLSM. A, two cutaneous pathways on skin. B, sampled between the pathways and showed irregular interlobular septum in hypodermic tissues. C, sampled from the cutaneous pathway and showed longitudinal interlobular septum towards the transport direction. D, scanned in the middle of (C) and showed the internal fibres of septum were distributed mainly longitudinally towards the transport direction. E, the fluorescently stained fibres within the cutaneous pathways in comparison with (F) (sampled from the left forearm as control without fluorescein transport). H and I, the 3D structures of the adventitia of (G). I, one plane in the yellow region of (H) and showed the longitudinal fibres were enriched in one side of the “conduit wall.” J, the fluorescently stained fibres within the adventitia of (G) in comparison with (K) (sampled from the left axillary vein as control without fluorescein transport). L, the fluorescently stained superficial tissues on the right atrial wall and pectinated muscles of atrial appendage. M, the intrinsic structures of the right atrial appendage and the superficial tissues (yellow box). N, the criss‐crossed fibres in the superficial tissues on pectinated muscles by micro‐CT. O, the fluorescently stained fibres within the right atrial appendage in comparison with (P) (sampled from another cadaver's right atrial appendage without fluorescein transport as control)

**Figure 6:**
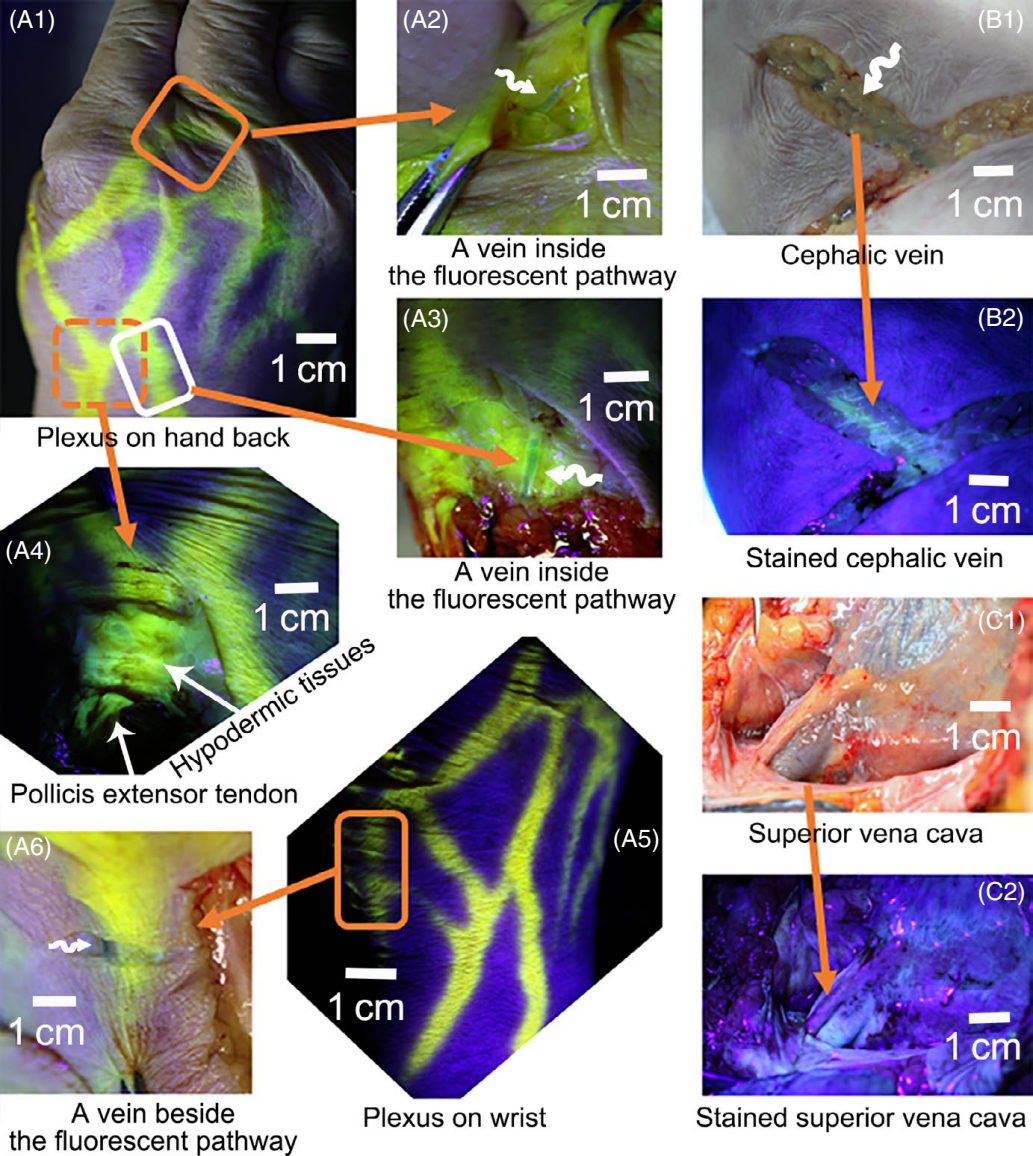
Illustration of the fluorescent pathways from LU11 of the 1 cadaver F/92. By the simulated heart beatings, the fluorescein from the fingertip visualized the fluorescent pathways that divided into brachial plexus on hand (A1) and wrist (A5). Interestingly, a branch went centrifugally to the bottom of the index and middle finger (A1, A2). The perivascular pathway was sometimes enveloped in the cutaneous pathway (A2, A3) or beside it (A6). The cutaneous pathways could contain diverse anatomic structures in one plane, including the dermis, hypodermic tissues, superficial fascia, deep fascia on the tendons of extensor pollicis longus, extensor pollicis brevis and pollicis longus (A4). B1, the hypodermic tissues under natural light of the fluorescently stained tissues of (B2). C1 and C2, the outer wall of superior vena cava, and right atrium was stained by the fluorescein

These fluorescent pathways were usually easily seen on the skin of hand and wrist, and they went deeper and vanished on the skin of the upper forearm and upper arm (Figures 3F,H,I and 4A). By layered dissection along the transport courses, the fluorescent pathways of hand and forearm were found to be composed of diverse anatomic structures, including the dermis, the hypodermis, superficial fascia on the tendons from the thumb or the muscles and loose connective tissues surrounding the veins (Figures 3F,H,I and 6A4). In the cubital fossa, the upper arm and axilla, the only fluorescently stained tissues were the perivascular connective tissues surrounding the cephalic and brachial vein, axillary sheath (Figures 3B,C, 5G and 6B1,B2). When the thorax was cut open, the outer walls of the superior vena cava, the superficial tissues on the right atrial wall and pectinate muscles of right atrial appendage were clearly stained by fluorescein (Figures 3L,K, 5L and 6C1,C2).

This transport course visualized by the fluoresce coincided with the findings with Gd‐DTPA in the three human cadavers of No. 11‐13 by MRI, especially in the body of a 7‐year‐old boy (Subject No. 13, Video S9).

### 3.3 The fluorescent pathways were neither blood nor lymphatic vessels verified by gross anatomic methods

The long‐distance fluorescent pathways were neither blood nor lymphatic vessels. Firstly, the fluorescent pathways were not the results of fluid through vascular lumen because the intraluminal blood taken from the right ventricular cavity and the opposite arm was not stained by the fluorescein during the whole experiments. Secondly, the width of the fluorescent pathways was much larger than that of the enveloped blood vessels and the lymphatics visualized by Chinese ink (Subject No. 14, 15; Figures 4A,B,E and 6A2,A3). Thirdly, the components of the fluorescent pathways in hand and lower arm contained diverse structures including the dermis, hypodermis and superficial fascia on muscles or tendons (Figures 3G,H and 6A1‐6). All these were not tubular structures.

### 3.4 The extravascular pathways visualized by 10 nm quantum dot microspheres

**Figure 7:**
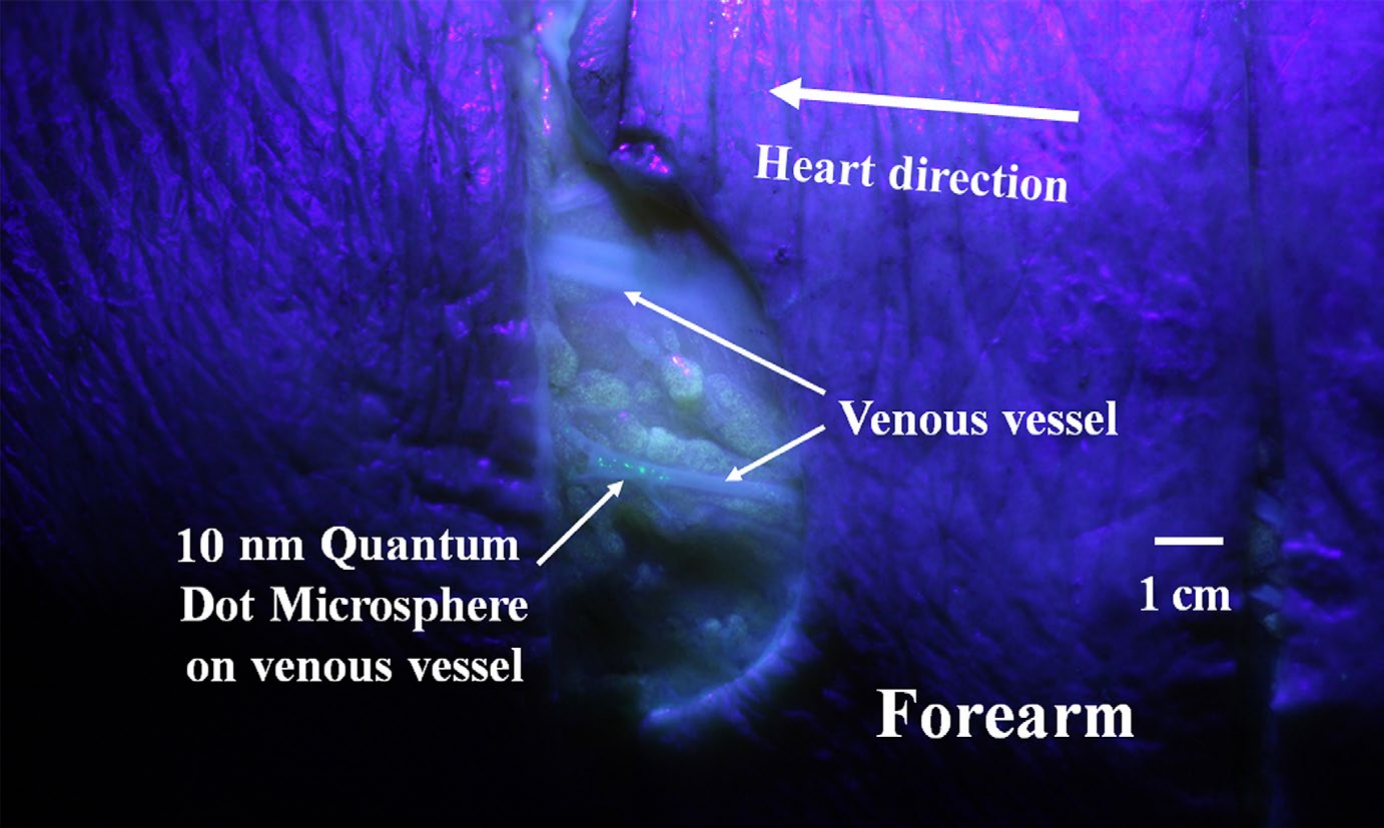
Illustration of 10 nm quantum dot microsphere on a venous vessel. After 2.5 h of compression on the heart, the 10 nm quantum dot microspheres from the right thumb can be found on a venous vessel in the forearm of the No. 16 subject

After the 2.5 hours of compression, both 10 and 110 nm quantum dot microspheres were found in the superficial tissues on the distal phalanx of right thumb of the No. 16‐19 subjects. Only 10 nm microspheres could be found on some venous vessels in the forearm of the No. 16 and 17 subjects (Figure 7). The number of the extravascular pathways visualized by 10 nm microspheres was much less than those by fluorescein sodium solution. There were no signs of 10 nm microsphere found on the atrial wall in the current experiment.

### 3.5 Histological analysis on the fluorescent pathways

Samples were taken from the skin of the hand and forearm, the cephalic vein and axillary sheath, the superficial tissues on the right atrial wall and pectinate muscles of right atrial appendage. By using methods of frozen fluorescence, Elastic van Gieson and H&E staining, respectively, the histological structures of the fluorescent pathways were composed of fibrous connective tissues (Figure 2,G1‐2,H1‐2). There were no continuous capillary conduits found in cross‐sections of the longitudinal fluorescent pathways by immunochemical staining against CD31 and D2‐40 (Figure 4,G1‐4,H1‐4). By TPLSM, all samples of the fluorescent pathways contained abundant longitudinal fibres along the transport long‐axis (Figure 5E,J,O), which coincided with our previous findings in human amputated lower legs.^6^ However, fluorescent fluid through arterial adventitia has not been seen here.

### 3.6 Three‐dimensional structures of the intrinsic fibre network of the fluorescent pathways observed by micro‐CT

By micro‐CT method,^9^ the intrinsic three‐dimensional architecture of the fluorescent pathways was clearly displayed (Figure 5C,D). The interlobular septum of the fluorescent cutaneous pathways in hand and forearm was identified as longitudinally oriented towards the transport long‐axis (Figures 3D,F and 5A,C,D; Videos S1 and S2). By contrast, the orientation of the interlobular septum outside the transport pathways was irregular (Figures 3E and 5A,B; Video S3). In venous samples, the adventitial fibres were mainly towards the vessel's long‐axis. The adventitial fibres were unevenly distributed in the annular adventitia and enriched in one side of vascular adventitia (Figures 3A and 5G,I; Video S4), which indicated that the adventitial fluid was unevenly transported along fibrous adventitia. The intrinsic structures of the superficial tissues on the pectinate muscles of right atrial appendage were composed of criss‐cross fibres by micro‐CT and TPLSM (Figure 5L,M,N,O; Video S5).

## 4 DISCUSSION

Beyond vascular circulations, we identified a cutaneous and perivascular fluid transport pathway that was composed of fibrous matrices of oriented connective tissues in human body. In line with our previous findings in the extremities,^6^, ^7^ the anatomic connection of these extravascular pathways from the distal end of thumb to the right atrium disclosed a profile of the systemic distributions of the long‐distance interstitial fluid transport pathway network. Our MRI results in volunteers indicated that more long‐distance extravascular pathways were initiated from the hypodermic entrances of other fingers and toes, usually from acupuncture points. Thus, their connections with the associated visceral organs or the structures need further verification in both the cadavers and volunteers. Considering that fibrous connective tissues are one of the four basic tissues and distributed in almost everywhere, we proposed that fluid in fibrous interstitial matrices is not totally fixed in *tissue gel* but transport partially along the extravascular pathways of oriented fibrous connective tissues under certain dynamic physical mechanisms, forming a network of long‐distance interstitial fluid transport pathways systemically throughout the human body or other animal bodies.

The long‐distance perivascular fluid transport was reported in several parts of the body, such as the perivascular spaces and the glymphatic pathways along the periarterial sheaths and the large‐caliber draining veins of mice brain,^2^, ^10^, ^11^, ^12^ the subcutaneous “perivascular‐spaces‐like” channel near the lymphatic and blood vessels of the abdominal skin of rabbits,^13^ the perivenous pathways along the adventitia of the lower extremity veins, inferior vena cava and into the pericardial cavity,^4^ some segments of small intestines and partial pulmonary veins of rabbits,^4^ and the perivenous pathways and the periarterial pathways in human lower legs.^5^ In fact, not only fibrous adventitia but also its surrounding general loose connective tissues were confirmed to be the perivascular pathways for fluid flow.^5^ These studies indicated that fluid around the entire vascular tree, including both venous and arterial adventitia, would be transported along fibrous adventitia and converge into the superficial tissues of the right atrium or other parts of the heart in physiological conditions. The revealed function of fibrous adventitia may trigger new inspiration on vascular biology (Figures 3A,B,C and 6B1,B2).

The long‐distance cutaneous pathways were also histologically identified as oriented fibrous matrices of connective tissues. Unlike vascular tree connects with the heart, the panorama distributions of the cutaneous pathways have not been understood yet. The cutaneous pathway from the distal end of thumb formed a plexus in hand and forearm (Figures 3I and 6A1,A5), which were composed of the millimetric interlobular septum of adipose tissues longitudinally assembled towards the transport long‐axis (Figures 3D,F and 5C,D; Video S1). By contrast, the dermic tissue beyond the cutaneous pathways was of irregular interlobular septum (Figures 3E,F and 5B; Video S3). Above the elbow level of the cadavers, the cutaneous pathways were not found and seemed to converge into the perivascular pathways along the cephalic and basilic vein, axillary sheath and the superficial tissues of right atrium eventually (Figures 3C,B,J, 5G,L and 6B1‐2, C1‐2). Whereas in volunteers by MRI, the cutaneous pathways from hand were found in the entire upper limb.^6^ The anatomic approach may not disclose the panorama distributions of the cutaneous pathways in volunteers.

The reasons may be due to the dynamic factors to drive interstitial fluid flow. In current anatomic study, only parts of the heart near the chest were motivated by the device. Both the cutaneous pathways in the upper arm and the periarterial pathways have not been observed in the cadavers. In physiological conditions, we have verified in one amputated lower leg patient, who took the tracer injection before the amputation, that there were four types of long‐distance extravascular fluid transport pathways at least: a cutaneous pathway, a perivenous pathway, a periarterial pathway and even a fibrous‐endoneurium‐perineurium‐epineurium pathway.^5^ Thus, it indicated there would be more extravascular fluid pathways driven by more dynamic factors in physiological conditions. Specific medical imaging technique was needed in alive human body to visualize the diverse long‐distance extravascular fluid transport pathways under the guidance of the disclosed anatomic structures in cadavers.

### 4.1 The intrinsic structures of the long‐distance extravascular fluid transport pathways with oriented fibrous matrices of connective tissues

The fibrous matrix of oriented connective tissues was identified to be the histological structures of the long‐distance interstitial fluid transport pathways as well as the same histological tissues of the interstitial matrix of extracellular matrices, fibrous cellular environment, perivascular spaces in brain, paravenous pathways, perivascular sheath, fibrous sheath, nerve sheath, epineurium, endoneurium, fascia, etc. Until now, the intrinsic three‐dimensional architectures of fibrous matrices and their long‐distance interconnections systemically throughout the human body have not been fully understood yet.

Revealed by micro‐CT, the millimetric tunica adventitia and longitudinal interlobular septum were identified to be composed of the micron‐sized fibres which were mainly oriented towards the transport long‐axis. By histological analysis, the long‐distance extravascular fluid transport pathways included different proportions of the hydrophilic collagen fibres and the hydrophobic elastic fibres (Figure 4,4,4), which indicated that each fibrous pathway contains some extent of hydrophilicity or hydrophobicity and may exhibit the mesoscopic features like interface chemistry/physics properties and surface interactions.^14^, ^15^, ^16^

### 4.2 An interfacial transport zone between the solid fibres and the surrounding gel‐like substances is responsible for fluid transport through the long‐distance fibrous extravascular pathways

Because the fibres and the gel‐like substances are fixed and cannot flow freely,^17^, ^18^ the only possible free space that allows fluid to transport is the interfacial clearances between fibres and gel‐like substances, which may be termed as “fiber/gel interfacial clearance” and abbreviated as “interfacial clearance.” Once the fluid is filled into the interfacial clearance, then a liquid film may be formed, which may be named as the “fiber/gel interfacial liquid zone” and abbreviated as “interfacial liquid zone.” Once the liquid flows in the zone along fibres under certain dynamic driving mechanism, then an “interfacial transport zone” may be set up.^19^, ^20^ Because the fibres are assembled longitudinally through a long‐distance fibrous extravascular pathway, the interfacial transport zone is of long‐distance characteristics. Besides, along the fibrous framework of oriented connective tissues, the long‐distance interfacial transport zones may be topologically connected and the “network of interfacial transport zones” in fibrous matrices of connective tissues throughout the whole body is created.

By using TPLSM, the abundant fluorescently stained fibres along the transport long‐axis were observed and the results of the fluorescent fluid transport through the long‐distance fibrous extravascular pathways, which clearly represented an imaging characteristic for the interfacial transport zones in fibrous matrices (Figure 5E,J,O). The network of interfacial transport zones may act as interstitial fluid transport pathways in the meshwork of fibrous connective tissues throughout the whole body.^19^ The present data were in agreement with our previous studies in the amputated lower legs.^5^

Illustrated by the 10 and 110 nm quantum dot microspheres, the pore sizes of the interfacial transport zones of the perivenous pathways may be <110 nm in the involved cadavers (Figure 7). The exact pore sizes of the interfacial transport zones of diverse fibrous connective tissues in physiological conditions need further studies.

### 4.3 The dynamic transport mechanism for the systemic extravascular fluid transport network

For the widespread fibrous matrices of connective tissues over the whole body, the dynamic transport mechanisms and regulating factors for the network of the long‐distance extravascular fluid transport have not been comprehensively studied. In the studies on the glymphatic system in brain, fluid transport through the perivascular pathways is related to the cerebrovascular pulsatility, body posture and perivascular astrocytic aquaporin‐4.^10^, ^11^, ^12^ In previous study on rabbits, fluid flow along venous adventitia into pericardial cavity was affected by the intactness and the wettability of the fibrous framework of the perivascular pathways as well as the periodic heart movements.^4^

As for the dynamic pattern of the long‐distance extravascular fluid transport pathways driven by the periodic to‐and‐fro mechanical movements of right atrium in this dynamic gross anatomic study, the following multiscale dynamic driving mechanisms from macroscopic scales to microscopic scales may be proposed. The repeated compressions of the mechanical device on chest generated a macroscopic driving centre, which was the right atrial wall and appendage near the chest here. At the microscopic scale, the long‐distance interfacial transport zones of fibrous extravascular pathways from the extremity ending to the driving centre provided a topologically connected interfacial clearances for interfacial liquid film throughout the oriented fibrous connective tissues. The periodic to‐and‐fro mechanical movements of the superficial tissues of the right atrium would act on the gel‐like fibrous tissues of the driving centre and lead to the periodical fluctuation of pressure. The periodical fluctuation of pressure will force the gel‐like tissues to compress and relax periodically, like a pump. The periodical compression‐relaxation of the pump played a role to “pull” the topologically connected interfacial liquid film in a long‐distance extravascular pathway. The exact roles of “driving‐center” on “interfacial‐liquid‐film‐transport” were complicated. We hypothesized that water molecules in interfacial liquid film would assemble a long‐distance link‐chain of hydrogen‐bonding or Van der waals' interactions to form “gearing‐chains,” which together with interfacial interactions may allow the long‐distance interfacial liquid films to be “pulled” towards a driving centre.^19^, ^21^, ^22^, ^23^ More experiments are necessary to verify the hypothesis of multiscale dynamic driving mechanisms, which is “fibro‐tissue driving‐transfer mechanisms for fluid transport,” named as “gel pump.”

As for the relationship between the long‐distance fibrous extravascular pathways and the vascular circulations, we verified in alive rabbits that fluid in ankle dermis would converge into the perivascular pathways, the venous and lymphatic vessels in the meanwhile.^4^ As fibrous matrix of connective tissues is the bed for capillaries, free interstitial fluid that was transported via the long‐distance fibrous extravascular pathways may easily enter capillary vessels by Starling forces in physiological conditions. In the current anatomic study, the intraluminal blood taken from right ventricular cavity and the opposite arm has not been fluorescently stained during the whole experiments, which indicated the long‐distance fibrous extravascular pathways were relatively independent from vascular circulation. In alive human body, the exact relation of the long‐distance fibrous extravascular pathways with vascular pulsation, Starling forces, interstitial pressure gradients, the electrical activities of nerves, the temperature and even electromagnetic force need further explorations.^18^

### 4.4 A potential relationship between the systemic fibrous extravascular pathways network and Traditional Meridians and Collaterals network

In accordance with previous findings in volunteers by MRI,^6^ if the injection point on hand or foot was not the acupuncture points, only the hypodermic diffusion and the perivascular pathways were displayed. Based on the orientations of the interlobular septum of adipose tissues, fluid outside the cutaneous pathways would spread through the irregular septum and cannot form an oriented cutaneous pathway (Figure 3E,F; Video S3) unless it converged into a site in the vicinity of vascular vessels and transported through the perivascular pathways. If an imaging tracer was injected into an acupuncture point of the fingers, toes, wrist and ankle, where anatomically connected with the long‐distance longitudinal interlobular septum, a cutaneous pathway would be visualized.

However, the transport courses of the fibrous extravascular pathways from the thumb were significantly individual in all subjects and different from the diagrammatic drawing of Lung Meridians in Meridian Atlas.^24^ The visualized fibrous pathways were not the proof for the Meridians & Collaterals network but could be an anatomic structure for interstitial fluid transport, mediated by which acupuncture signals from an acupuncture point can be transmitted into an associated visceral structure hypothetically.

In summary, fibrous matrices of connective tissues are thought to contain irregular three‐dimensional matrices and act as glue for cells to attach to matrix. Our data verified the structural framework of an oriented fibrous connective tissue was composed of the multi‐layered, longitudinally assembled and intertwined micron‐sized fibres from an extremity ending to an associated visceral structure. Upon the network of topologically connected interfacial transport zones along the long‐distance, oriented and orderly assembled fibres, interstitial fluid can be transported in the meshwork of fibrous connective tissues, named as “fibro‐tissue interfacial transport.” These data strongly suggest that interstitial fluids in the extracellular matrices are not fixed in *tissue gel* but systemically transport throughout human body and enter blood vessels and initial lymphatic vessels eventually by the Starling forces. We hypothesized that: (a) the systemic interstitial fluid transport together with the cardiovascular system and lymphatic system comprises three principal components of circulatory system in animals and humans. (b) Unlike blood and lymph through vascular conduits, interstitial fluid is transported via oriented fibrous connective tissues openly without vascular walls of endothelium, contacts with the parenchymal cells and the surrounding tissues along the way of the long‐distance transport towards various driving centres and in the meanwhile exchanges with circulatory and lymphatic system in physiological conditions, named as “interstitial fluid circulation.” The exact relationships of the systemic interstitial fluid circulation with vascular systems, nervous system and even all other anatomical or functional biological systems need to be further clarified. We also hypothesized that the revealed fibro‐tissue interfacial transport pathways provided a long‐distance transduction pathway for extracellular signals or other bio‐signals (including acupuncture signals) from the extremities into the associated visceral structures or among cells, various tissues and organs. Future studies on the kinetics/dynamics, anatomic distributions and physiological/pathophysiological functions of the interstitial fluid circulatory system would bring more exciting findings in life science. Drawing human anatomic atlas of the oriented fibrous pathways and developing the associated imaging technique would be key bridge between basic medical science and the clinical diagnostic and therapeutic applications in these interdisciplinary fields.

## CONFLICT OF INTEREST

The authors declare no competing financial interests and have no conflicts to disclose.

## AUTHOR CONTRIBUTIONS

HyL conceived the conception and designed the experiments. HyL, NlW, DZ, YpC, LX, XxW, YyK, QL and SS performed the gross anatomic experiments. HyL and CqY performed histological analysis of all tissues. LrD, HyL and DH performed the TPLSM experiments. HyL, FsJ, FW and ZA analysed the micro‐CT data. HyL, MC, WtL and HL analysed the MRI data. CM proved the usage of human cadavers. HyL and YjY analysed the interfacial transport pattern. HyL wrote the manuscript. LjS made English corrections. All authors contributed to scientific discussions of the manuscript.

## Data Availability

The data that support the findings of this study are available on request from the corresponding author. The data of the donated cadavers are not publicly available due to privacy and ethical restrictions.
