# Supplementary material for: An extravascular fluid transport system based on structural framework of fibrous connective tissues in human body
Source: Cell Prolif. 2019 Aug 1;52(5):e12667. doi: 10.1111/cpr.12667 (PMC6797508; doi:10.1111/cpr.12667)
Supplement: Supplementary file 10 [file CPR-52-e12667-s010.docx]

**Supplemental data**

Video 1. A three-dimensional view of the hypodermic interlobular septum of the cutaneous pathway sampled from forearm by micro-CT. The interlobular septum was distributed longitudinally toward the long-axis of the cutaneous pathway.

Video 2. A three-dimensional view of the hypodermic interlobular septum of the cutaneous pathway by high resolution micro-CT. The internal fibers of septum were distributed mainly longitudinally toward the transport direction.

Video 3. A three-dimensional view of the hypodermic interlobular septum beyond the cutaneous pathway sampled from forearm (Fig. 3E) by micro-CT. The interlobular septum was distributed irregularly.

Video 4. A three-dimensional view of the adventitial fibers. The fibers were distributed longitudinally toward the long-axis of the vessel and enriched in one side of the “conduit wall”.

Video 5. A three-dimensional view of the crisscrossed fibers. The crisscrossed fibers were in the superficial tissues on the pectinated muscles of right atrial appendage.

Video 6. A three-dimensional view of the cutaneous pathways and the perivascular pathways of the subject 6 in figure 2. The cutaneous pathways and the perivascular pathways originating from HT9 were visualized in hand and forearm.

Video 7. A three-dimensional view of the angiography of the subject 6 in figure 2. The superficial veins in hand and forearm were visualized by intraluminal Gd-DTPA.

Video 8. A three-dimensional view of the cutaneous pathways and the perivascular pathways from ST45 on index toe of the subject 11 in figure 2. The cutaneous pathways and the perivascular pathways originating from ST45 were visualized in lower leg.

Video 9. A three-dimensional view of the upper arm of the 13^th^ cadaver of M/7. By contrast the left upper arm, the cephalic vein, basilic vein and axillary vein of right arm were enhanced by Gd-DTPA from the thumb fingertip. Under the axillary vein, it showed that a segment of subclavian artery was enhanced but we cannot confirm that the subclavian artery was stained by fluorescein in other cadavers.
